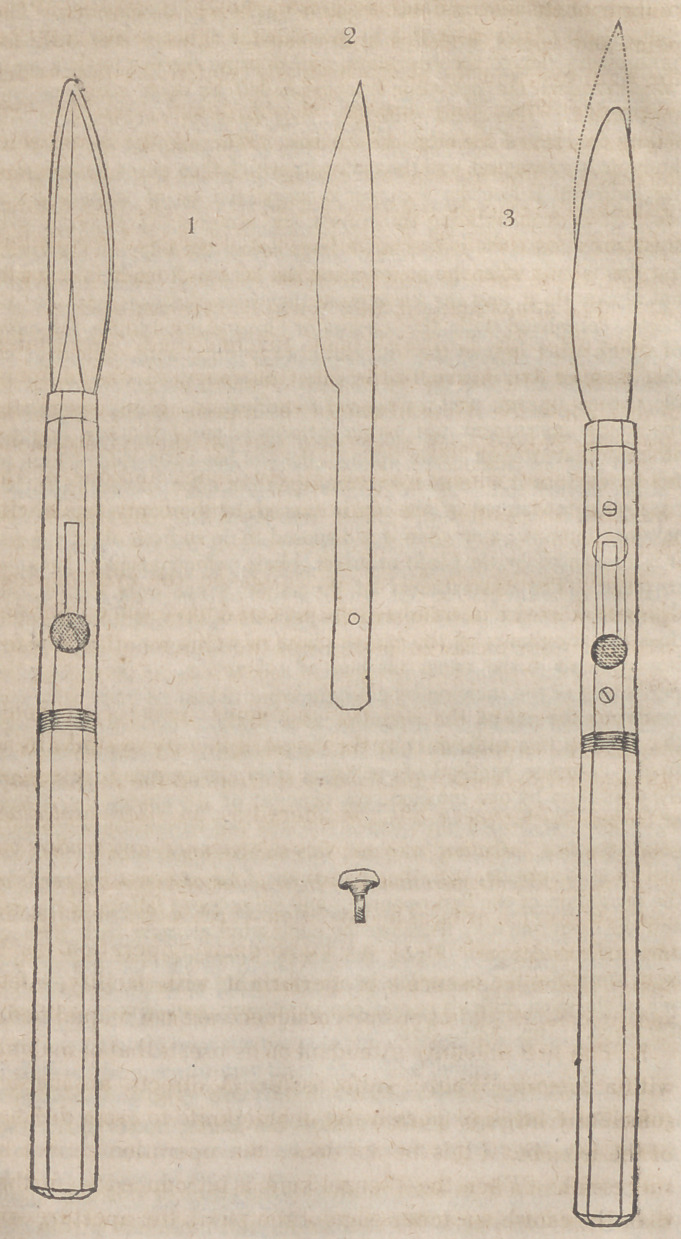# Observations on Mr. Guthrie’s Double Cataract Knife

**Published:** 1828-08

**Authors:** 


WESTERN JOURNAL

OF THE

### MEDICAL & PHYSICAL SCIENCES.

AUGUST, 1828.

### ESSAYS AND CASES.

### Art. I—*Observations on* Mr. Guthrie's *Double Cataract Knife.*

BY THE EDITOR.

Mr. Guthrie, the inventor of this instrument, is one of the Surgeons of the "Royal Westminster Infirmary for the cure of Diseases of the Eye." In the second edition of his valuable Lectures on the operative Surgery of the eye, published at London in 1827, he describes the instrument with its application and advantages, in the following words:

"In order to avoid some of the difficulties attendant on the division of the cornea, Dr. F. Jager, of Vienna,* has proposed a double knife, one rather smaller than the other, attached by a button screw, yet capable of sliding upon it by pressure with the thumb, for the purpose of completing the incision of the cornea, when the punctuation is completed by the larger and lower one. It is to be introduced in the same way as Beer's knife, not parallel, but nearly perpendicular to the cornea; and afterwards carried across the eye, exactly like the single knife, with the blunt edge, or back, following an ideal line drawn directly from one side to the other, and with the external side of the fixed blade parallel to the iris, at the usual distance from the junction of the cornea with the sclerotica. When the point of the greater knife has transfixed the cornea at the inner side, pressure is to be made on the button head with the thumb; the smaller knife is, in consequence of the pressure, pushed forward, while the greater keeps the eye steady, until the operation is finished. A needle is then introduced to lacerate the capsule, and the opera-

*See Loudon's Short Inquiry into the Principal Causes of the Unsuccessful Termination of Extraction by the Cornea. London, 1826.

tion concluded by gentle pressure on the ball, if necessary, to displace the lens from its situation.

"In employing the double knife, we may use it as we would a Beer's knife. After the counter-punctuation is made, we have a support for the ball in the greater knife, while the cut is performed by the smaller. There is no necessity for dragging forward the eye to enable the operator to complete the incision."

The advantages above described, have not all been observed in the trials I have made with the instrument, and I suspect they will be found to be more specious than real. I am not, however, as yet competent to give a decided opinion either for or against the affirmative one of Jager, or of our author, Mr. Loudon.

In denying, however, to this instrument, the merit which has been attributed to it, I am willing to concede that of its being the foundation for the construction of another, which I believe to be more perfect—to be capable not only of preventing nearly all the difficulties and dangers which occur in the extraction of the cataract, but of obviating them when they have occurred. either from accident, or any defect in the operator. It is a double instrument, one part being a cataract knife of the shape of Wenzel's, the other a silver blade of the same form, but larger and blunt. (See Plate.) The sharp steel knife is attached to the silver blade by a button screw in the handle, which admits of the knife being pressed forward in the same manner as Jager's; whilst it is so nicely and closely fitted to the silver blade as to form one instrument, neither the point nor the edge catching when the finger is pressed along it. An opening is to be first made in the cornea, with a common large Wenzel's knife (which I prefer to Beer's;) and of such a size as to admit the double instrument, being the first step of the punctuation: the knife being withdrawn the aqueous humour escapes, and the iris is pressed forwards against the cornea, the eyelid being allowed to fall over it. If any protrusion of the iris should take place at the opening in the cornea, the protruded part will return to its place on gently rubbing the eyelid with a silk handkerchief, a piece of sponge or the finger. The eyelid is now to be raised, and the double instrument introduced at the opening, the silver blade being next the iris. The silver point being larger than the steel one, easily raises the cornea and presses back the iris; so that by alternately raising and depressing the point of the instrument, it is readily carried across the eye in front of the iris and pupil, until the silver point touches the inside of the cornea, either immediately opposite the point of entrance, or as much above or below as the operator may think fit. The thumb, which has hitherto been resting on or near the button screw, is now made to press it forward, and to protrude in consequence the sharp steel blade through the cornea, when the instrument readily cuts its way out, and completes the section of the cornea.

The principle on which this instrument is used is diametrically opposed to all others. The great object of Wenzel, Beer, and Jager, and of all operators, is to prevent the evacuation of the aqueous humour, until the knife has passed across the eye through the cornea at the opposite side, and has begun to cut its way downwards. The great object of this method is to evacuate the aqueous humour, as a preliminary step to the operation, and to bring the eye to that state which renders the operation recommended by those surgeons impracticable. The great difficulty they have to encounter is the falling forward of the edge of the iris, at the moment the aqueous humour is evacuated, and the necessity which then arises for bringing the knife out before the incision is completed, or of wounding the iris. It is true, rubbing the cornea, as directed by Wenzel, will sometimes cause the iris to move from before the edge of the knife, but this is only when the punctuation has been well made; for it will not always do it, and the knife must then be withdrawn, and the incision completed with the scissors or blunt-pointed knife, the edge of which often injures the iris, and always cuts with difficulty; so that three or four cuts will be required, leaving the general edge of the cornea uneven and indisposed to adhesion. In my operation, the double instrument not being introduced until the eye is quiet after the first attempt made upon it, the iris has little disposition to fall forward, as it is termed, or turn upon the edge of the knife, because this movement of the iris is caused by the evacuation of the aqueous humour; and when it is disposed to do so from other causes, it is prevented by the blunt or silver blade, which keeps it back in its place. The unsteadiness of the patient's eye only delays, but does not otherwise interfere with the success of the operation, neither does the toughness of the cornea, both of which are often serious impediments in the usual method of extraction, as preventing the perfecting of the incision by a single introduction of the knife, and rendering the use of the round-pointed knife necessary to enlarge the opening to a sufficient size for the passage of the lens through it. In the common method of operating it is dangerous to complete the operation by one incision, on account of the spasmodic action of the muscles, which often expels the lens and vitreous humour with great violence. By my method, this sudden spasmodic action has had time to subside, with the evacuation of the aqueous humour, by the formation of the first opening, and subsequent falling of the lid, and the operation may therefore be completed at once with little or none of this danger. Upon the whole, I consider that this instrument simplifies the operation in a remarkable manner, and renders one of the most difficult operations in surgery, one of very easy performance." pp. 343—346.

The following plate, copied, with some slight alterations, from Mr. Guthrie's book, exhibits his knife, under three different aspects.

Figure first shows both blades;—the steel or cutting blade pointed, resembling a Wenzel knife, and included within the

2
1
3

contour of the silver blade or guard, which is obtuse, at the point and edges, and lies underneath. This is the knife for the right eye, and the blades have the relative position, which is proper when they are to be introduced into the eye. Figure second exhibits the cutting blade of the same side, detached. The hole in the shank receives the screw point of the knob, which is represented below. Figure third shows the knife for the left eye; with the cutting-blade projected, as it is, at the time when the section of the cornea is completed. The silver blade in this figure is underneath.

Soon after Mr. Guthrie's book reached the United States, I requested Dr. Hays, of the Eye Infirmary, at Philadelphia, to put it into the hands of one of the ingenious cutlers of that city, Mr. Rohrer, and have two sets of these instruments made for me, which was obligingly done; and one of the objects of this paper is to state the results of my trials with them.

Appropriating one set to operations on the eyes of dead animals, I used it a number of times, and have since operated with the other on three patients of the Cincinnati Eye Infirmary.

From the language of the ingenious inventor, it could scarcely be supposed that the least difficulty would attend the use of this knife, and in his experienced hand, this may, perhaps, be the case; but considered as an *instrument which simplifies the operation in a remarkable manner, and renders one of the most difficult operations in surgery, one of very easy performance;* in other words, as an invention which so far obviates the the difficulties attendant on the ordinary operation, as to enable the inexperienced to perform it with facility, safety and success, its claims on our confidence are not unqualified.

I. The first difficulty attendant on its use, is that of making, with a common Wenzel knife, as Mr. G. directs, an incision sufficiently large to permit the double knife to cross the disk of the iris; but if this be not done, the operation cannot be successful. When the Wenzel knife is introduced no further than the centre, or inner edge of the pupil, the aperture will be too small to admit the Guthrie-knife; and, if, to increase the dimensions of the incision, the Wenzel knife be made to traverse the iris, the operation might, in general, be safely completed without withdrawing that instrument: in other words without Mr. Guthrie's knife. Those, therefore, who are about to use his knife, should do one of three things, in reference to this difficulty. 1. In withdrawing the Wenzel knife, the handle should be rotated on the point, by which the cornea will be cut to a greater extent, than by the mere puncture; but as the aqueous humour will begin to escape, as soon as the rotation commences, this method may not be without its dangers to the iris. 2. After withdrawing the knife, one of Mr. Guthrie's silver guards (the cutting blade being detached) may be introduced; and, while the iris is thus protected, the cornea, may be cut, by successive strokes with an iris knife. This operation is safe, but tedious; and renders the lips of the wound more or less irregular. 3. Instead of using the Wenzel knife, one much less tapering might be made; which from its great breath would make a sufficient incision by the time the point had reached the outer margin of the pupil. I perceive no difficulty either in the construction or use of such a knife; and, it would effectually obviate one of the greatest objections to the new instrument.

II. Having succeeded in introducing the Guthrie-knife, the inexperienced hand encounters a new difficulty, which nothing but practice can surmount. While the silver blade is pressed forward and kept firmly in its position, across the anterior chamber, the cutting blade is to be projected. In doing this the silver blade has a tendency to recede; and without proper care we shall move it backwards, instead of moving the other forwards. If the first incision should not have been sufficient, to permit the point of the silver guard to reach the cornea, on the opposite side, this recession will almost inevitably occur. It can only be prevented by pressing the point of the guard against the cornea, where the second puncture is to be made, sustaining that part by the middle finger of the left hand; placed over the caruncula lachrymalis. In this manner the guard may be kept firmly and steadily in its place, while the cutting blade passes across the eye, and pierces the cornea on the inner side. If on introducing the instrument, the preparatory incision should be found to be too small, it should be withdrawn, and the cut made larger, with the scissors, or preferably, in the manner pointed out above; and the operator should not attempt to enlarge it by projecting the cutting blade, and at the same time moving the guard across the eye. The two motions, with the same fingers, could not be performed with success. Hence, as it respects the whole operation, the great importance of a free initial puncture.

III. When the muscles of the orbit are prone to convulsive action, the eye ball cannot be kept from rolling, without a degree of pressure, that causes more or less *prolapsus iridis*, at the moment when we attempt to introduce the double knife. I am aware that in cases of spasmodic action, the vitreous humour is liable, under the old method, to be thrown out, and that Mr. Guthrie's method will diminish that liability; but in doing this, it evidently, produces a different difficulty, that which I have named, and in some cases this may be more momentous than the one which it obviates.

IV. The passage of the instrument between the cornea and iris, both relaxed and quite in contact, is not without its difficulties; for each of them, in turn, is disposed to catch and wrinkle on the silver point, and the latter to float around the obtuse edges of that blade, so as even to be in danger of injury from the steel knife.

V. The thickness of the silver blade, if it be so strong as not to bend, is such, that if the first incision be not made extremely near to the sclerotica, the cut of the steel blade, will be so far towards the apex of the cornea, that the central part of the eschar may, to a certain degree, interfere with the vision of the patient.

VI. After the perfect evacuation of the aqueous humour, from both chambers of the eye, through the initial incision, and the consequent collapse and relaxation of the cornea, that membrane probably becomes tougher, and more difficult to cut; so that the remainder of the incision cannot be executed with the same facility, as in the ordinary operation.

I have used Mr. Guthrie's knife three times. The first case terminated unfortunately. I will state the manner, as it involves several of the difficulties which I have pointed out; and may possibly be the means of preventing a similar result, in the first trials which other operators may make with his instrument.

The patient, a lad 14 years old, laboured under congenital cataract. I had operated on him according to Mr. Sanders' method and found the body of the lens unabsorbed and soft. Immediately after the operation it suffered the usual increase of opacity, and induration. At the end of several weeks it had undergone no perceptible dissolution, and a portion of it appeared to plug up the pupil. As the eye was well formed, the operation of extraction seemed quite practicable, and I determined on using Mr. Guthrie's knife. The initial cut was made with a large Wenzel knife, introduced until the point was near the nasal edge of the pupil. On withdrawing that instrument and attempting to insert the Double knife, I found the spasmodic action of the muscles of the globe (as is usual in congenital cataract) so considerable, as to require more than the ordinary pressure to keep it steady; and this pressure united with that of the convulsed muscles, protruded the iris into the incision, and against the cornea to such a degree, as rendered this part of the operation exceedingly difficult. By gentle efforts, however, the instrument was insinuated, without injury to the iris, as far as the Wenzel knife had been inserted, when the puncture would not suffer it to advance. I had then either to withdraw it and enlarge the incision, or to attempt its enlargement, by projecting the steel blade. As the tendency to a protrusion of the iris was so considerable as to give the appearance of difficulty in executing the former, I decided on performing the latter. I accordingly attempted to project the cutting blade, and as fast as it should enlarge the opening to push forward the silver guard; but from the unsteadiness of the globe, and the complex movements required from the same fingers; the cutting blade instead of traversing the cornea, at that time closely in contact with it, pierced that membrane a little below its centre, and cut its way out. Thus, instead, of a semicircular cut, extending half round the cornea, parallel to its margin, I had a curved incision, commencing at the usual point, and terminating in front of the lower part of the pupil. Thro' this incision the lens was discharged without violence. It was followed by a moderate portion of the vitreous humour. The wound healed, and the anterior chamber was properly replenished; but the eschar as might have been expected renders the cornea opake in the centre, and prevents the admission of light. I now believe, that had I withdrawn the double knife, and enlarged the aperture, in the manner, which I afterwards adopted, the result might have been favourable.

The subject of the next case was a man 50 years of age, whose eye was was steady. The initial puncture was made as before, and enlarged as follows—detaching the steel from the silver blade of another Guthrie-knife, I introduced the latter; and used it at once as a defence to the iris, and a director, for a small scalpel, with which I continued the incision, until it would freely admit the double bladed knife. This I then passed, without much difficulty, across the anterior chamber; and pressing the point against the cornea of the opposite side, supported by a finger of the left hand, I completed the incision without difficulty, and the case terminated favourably.

The subject of the third operation was a young man. Having to operate on the right eye, I determined to make the incision upwards. The cornea was punctured as before, but from the great tendency of the globe of the eye to role upwards I found it quite impossible to enlarge the aperture in that direction, and was obliged to operate downwards. From the unsteadiness of the eye, the lengthening of the cut was a slow operation, but I at length succeeded, and was enabled to push the double knife across the eye, and finish the operation without accident.

In both these cases, the incision was further from the junction of the cornea and sclerotica than is desirable, in consequence of the thickness of the silver blade; and I am disposed to think that an iron blade, which might be made much thinner, without bending, would be preferable.

From this review of the difficulties connected with the practical application of Mr. Guthrie's knife, I am compelled, to believe, that its value is less than he has supposed. It must be recollected that he offers it as a substitute for the knives of Beer and Wenzel, on the ground of its simplicity and safety. His recommendation, in fact, amounts to this, that while repeated trials, many of them disastrous to the eye on which they are made, have been found indispensable to a competent degree of skill in the old method; the new one is so easy, as to render long experience unnecessary to a successful result. But, although intrinsically a valuable method, I am disposed to think that a course of practice, little short of that which would make the operator familiar with the old method, is absolutely necessary to any tolerable perfection in the new.

After all, however, Mr. Guthrie's knife is not without its advantages. Whenever, from any cause, the further introduction of the common cataract knife would endanger the iris, and its withdrawal lead to a failure in the operation, Mr. Guthrie's knife offers an admirable resource; and cases of this kind may frequently occur. But its value when the muscles of the eye are powerfully convulsed, is more disputable, on account of the pressure of the iris against the wound in the cornea. It may tend to preserve the vitreous humour, but in the attempt to introduce it under such circumstances, there is danger of injuring the iris. On the whole it seems to me, that Mr. G. has overlooked the objections to his method—no uncommon thing in men of inventive genius, and sanguine anticipations.

In concluding I may say a word on the means of preserving his knife from oxidation. Whenever two metals of different degrees of oxidability are suffered to remain nearly in contact, with a fluid between them, a galvanic process is set up, and one of them will rust. Thus when the steel blade of the Guthrie knife is oiled and suffered to remain in connexion with the silver guard, it becomes speedily oxidated, as I have experienced; the two blades should, therefore, be kept asunder with cotton or soft paper.

## Figures and Tables

**Figure f1:**